# Effects on Facial Growth Following Masseter Muscle Resection in Growing Rats—A Systematic Review

**DOI:** 10.3390/ani13101680

**Published:** 2023-05-18

**Authors:** Georgia Kotantoula, Ioannis A. Tsolakis, Ioannis Lyros, Miltiadis A. Makrygiannakis, Christina Kanareli, Maria Dalampira, Apostolos I. Tsolakis

**Affiliations:** 1 Department of Orthodontics, School of Dentistry, National and Kapodistrian University of Athens, 11527 Athens, Greece; 2 Department of Orthodontics, School of Dentistry, Faculty of Health Sciences, Aristotle University of Thessaloniki, 54124 Thessaloniki, Greece; 3 Department of Orthodontics, Case Western Reserve University, Cleveland, OH 44106, USA; 4 Private Practice, 75015 Paris, France; 5 Private Practice, 54124 Thessaloniki, Greece

**Keywords:** mandibular growth, masseter muscle resection, mandibular length, ramus height, rat

## Abstract

**Simple Summary:**

Facial symmetry is a crucial determinant of facial appearance; treatment of asymmetry can be challenging. When it comes to the mandible’s development the muscles play a significant role in the development and growth direction of the mandible. However, it is unclear how the masticatory muscles contribute to early facial development, nor do we know to what extent they are involved in asymmetry. Our objective was to investigate the impact of masseter resection on mandibulofacial growth by reviewing the data of previously published studies on rats up to October 2022. The results of this study provide evidence that experimental masseter muscle excision in developing rats causes atrophic changes in the angular process, asymmetry in the maxilla, and overall shortening of the mandible. These changes occur as a direct outcome of the removal of the masseter muscle. Due to the mandible’s posterior rotation, muscular excision did cause specific articular alterations in the temporomandibular joint. Lateral X-rays of the mandible of the rats who were part of the experimental group revealed that their mandibular plane angles were higher than those of the control group.

**Abstract:**

An individual’s facial appearance is heavily influenced by facial symmetry. In the asymmetric mandible, periosteal apposition and endochondral ossification in one of the condyles may stimulate asymmetric growth of the body. Our aim was to review the impact on the growth following masseter resection. Relevant studies up to October 2022 were retrieved from PubMed, Scopus, and Web of Science. The PICOS method was utilized to determine eligibility, and the SYRCLE risk of bias tool was utilized to provide an estimate of potential bias. A predetermined algorithm was used to search the databases. The results of our systematic review of seven studies indicate that the masseter muscle strongly impacts craniofacial growth and development. Resection of the masseter muscle significantly reduces the sagittal and vertical development of the jaw in rats. In addition, the masseter muscle excision influences the mandibular morphology, including the condylar area, angle, and development direction of the jaw.

## 1. Introduction

The development of the mandible and condyle is determined by biomechanical factors in masticatory function [1]. Tsolakis et al. [2] demonstrated that the condyle is not the major factor that governs and guides mandibular growth since condylectomized mandibles of rats continued to participate in mastication, deglutition, and respiration during their growing period. The functional matrix theory proposed by Moss suggests that the muscles of the mandibulofacial system play an important part in craniofacial growth [3]. Previous studies have shown that a reduction in the force exerted by the masticatory muscles has an effect on the production of the mandibular bone and the proliferation of chondroblasts [4]. The function of the masticatory muscles has been altered using a variety of techniques, such as denervation or resection of the muscles, extraction or grinding of healthy teeth, the placement of devices in the mouth to change the way food is masticated, and changes to diet consistency [5,6,7,8,9].

Nevertheless, the conclusions following animal experiments have been unclear as to the extent of the genetic or environmental (functional) contribution to the growth and development of the jaw bones [10]. Animal studies and clinical investigations have examined the relationship between masticatory muscle function and craniofacial development. Previous studies reported that mandibular growth is heavily dependent on the growth of the condyles, which can be impacted by factors such as genetics, hormones, environment, systemic disorders, and stress [11]. It has been shown through research that the performance of the muscles of mastication is a contributor to the quality of bone that forms in the growing mandible [12].

Asymmetry of the mandible is a frequent clinical issue affecting the craniofacial area. Understanding the connection between the craniofacial morphology and voluntary muscle activity is crucial for treating individuals with mandibular deviation in order to evaluate the mandibular growth and potential impacts to the orthodontic or surgical treatment. Treatment for cases of asymmetry has proven to be challenging [2,13].

It is important to note that a person’s facial appearance may have an effect on their self-esteem and quality of life [14]; for this reason, an orthodontist will attempt to diagnose facial malformations at an early stage and then will proceed to treat them [15]. Functional deviations are causes of asymmetric craniofacial growth, since the temporomandibular joint and the alveolar bone are both subjected to the forces generated by the process of mastication.

Normal mandibular growth requires sufficient masticatory muscular strength throughout growth, and masticatory muscle function is a determinant of bone quality in the growing mandible. The mechanism by which changes in the mechanical stress affect the cartilage metabolism is still unclear. During mandibular growth, the condylar cartilage serves as a regional adaptive growth site [16]. It is believed that the mandibular cartilage is extremely adaptable to biomechanical pressures. Condylar cartilage functions as a component of the articulatory joint and as the mandible’s principal growth location. Furthermore, condylar cartilage also varies from other cartilaginous tissues in its histological structure, reactivity to biomechanical stress and humoral stimulation, and mechanisms of proliferation, differentiation, and calcification [17,18].

To retain its correct physical and biological qualities, articular cartilage requires mobility and joint loading. In reaction to damage or extreme mechanical stress, the number of cartilage cells decreases. Due to the absence of nerves, blood arteries, and self-repair in articular cartilage, chondrocytes are able to compensate for this deficiency due to the presence of growth factors. The development and growth of the mandibular condyle significantly affect the craniofacial skeleton formation and functionality of the temporomandibular joint (TMJ). Anterior disk dislocation of the TMJ may result in condylar degeneration or gradual resorption, which may impede mandibular growth if left untreated in children, a study suggested [19]. In the field of evaluating TMJ, magnetic resonance imaging (MRI) is regarded as the gold standard due to its unmatched advantages as a noninvasive radiation-free technology with good tissue contrast and the capacity to measure joint effusion [20].

According to the findings of several pieces of research, the severity of the jaw deformity has a strong relationship to the amount of condylar resorption, the degree of disk deformity, and the extent of the disk displacement [21]. Even though several studies have demonstrated that individuals with asymmetry are more likely to have condylar size and height regression, there are still certain limitations in studies of humans, such as a small sample size or mismatched study groups [22].

An asymmetrical mandible can be generated in a rapidly growing experimental animal. Unilateral masticatory muscle resection has been observed to result in an asymmetric morphologic alteration of the mandible in growing experimental animals [2,6,12]. As a model for scientific investigation, the rat is the animal of choice at the moment, but rabbits were also utilized in earlier studies [6,23]. Previous research has also employed rhesus monkeys [24,25] as an experimental animal model to examine the effect of masseter muscle excision on facial development.

The current study aims to gain a greater understanding of craniofacial growth due to masticatory hypofunction by examining possible structural TMJ alterations resulting from masseter muscle resection. The purpose of this review was to thoroughly evaluate the quality of the evidence that is now available in animal research regarding the effects (macroscopic, quantifiable, and dimensional changes) that occurred after masseter muscle resection in mice.

In compliance with the PRISMA-P recommendations, a particular protocol was created and put into use [26]. We complied with the Cochrane Guides for Systematic Reviews of Interventions and the PRISMA statement [27,28].

## 2. Materials and Methods

### 2.1. Eligibility Criteria

The eligibility criteria for our study were developed in accordance with the PICOS standards and are shown in Table 1. The relevant studies involved healthy animals undergoing unilateral or bilateral masseteroctomies.

### 2.2. Information Sources and Search Strategy

Utilizing the Medline Database up to October 2022, a literature search was conducted. Three databases (PubMed, Scopus, and Web of Science) were utilized to find all pertinent studies regardless of language, publication date, or publishing status (Table 2). In addition, we thoroughly combed through the cited works in order to identify more studies. Excluded were studies that were not comparable, reviews, systematic reviews, and meta-analyses.

### 2.3. Study Selection

The obtained records were independently evaluated by both the authors, G.K. and I.A.T. The eligibility of all recovered records was determined using the same methodology, despite the fact that they were not blinded to the identities of the authors or the findings of the research. Discussions with the co-author A.I.T. cleared up all uncertainties.

### 2.4. Data Collection

Data extraction was carried out separately by the same author, and any discrepancies were once again settled through discussion or consultation with the co-author A.I.T. The following information was recorded using predetermined and pre-piloted data collection forms: bibliographic information about the study, information about the research’s design and eligibility verification, information about the participants’ characteristics and the masseter intervention, features of the outcome measurement, and the findings.

### 2.5. Risk of Bias in Individual Studies

Both G.K. and I.A.T. independently evaluated the risk of bias using the SYRCLE risk of bias tool (2014) [27] and the standards established by Higgins and Green [29].

### 2.6. Summary Measures and Shaping of Results

If deemed possible, data on craniofacial morphology were to be combined using the random effects method for meta-analysis [30].

Meta-quantitative analysis of the data synthesis was not carried out as initially expected due to the lack of sufficient data on outcomes and the variances across the different approaches.

## 3. Results

### 3.1. Study Selection

**Figure 1:**
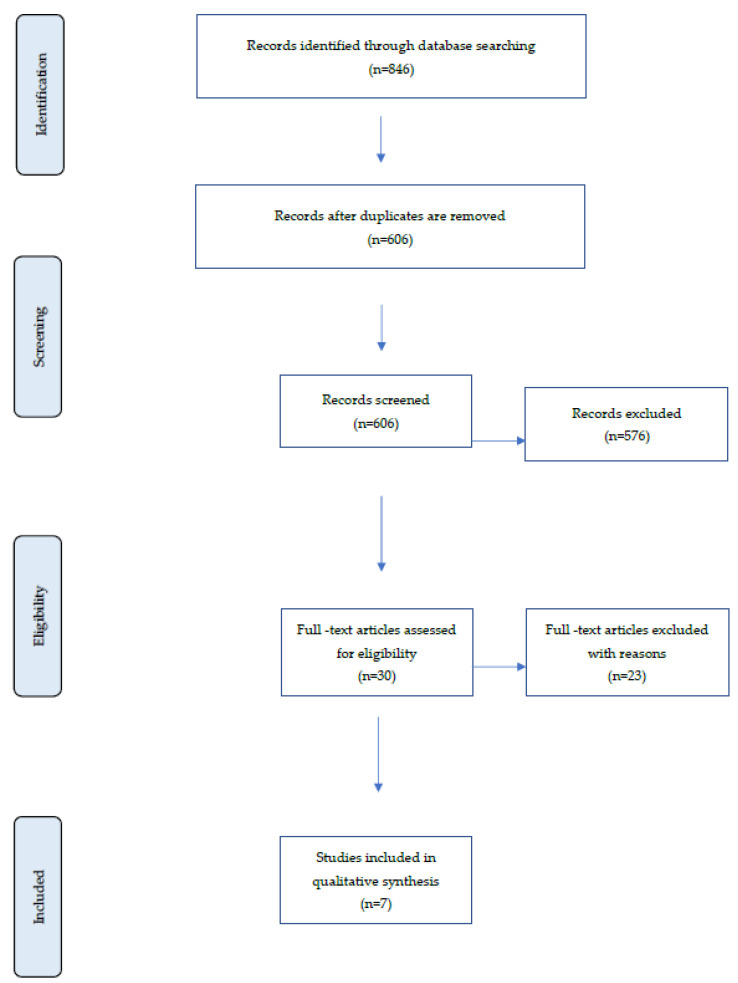
Flowchart of records during the assessment process.

Figure 1 outlines the reviewing process’s progression. From the initial search, 846 records were selected for further review. From these, 606 were eliminated as duplicates, and 576 were eliminated after title and abstract evaluation. From the remaining 30 studies, 23 were excluded due to absence of masseter muscle intervention with resection, the absence of radiographic evidence, and/or referred to species other than rat. Finally, addressing alveolar bone changes following alternation in masticatory function in the rat, seven full-text records comprised the systematic review [12,31,32,33,34,35,36].

### 3.2. Study Characteristics

Summary data from the selected studies are shown in Table 3. Most of the papers conducted their experiments on Wistar strain growing rats, while one work also made use of Sherman strain rats [33]. In addition, two studies [12,35] only used female Wistar rats, while Miyazaki et al. [34] did not describe the sex distribution of their rat sample. As a technique for assessment, cephalometric radiographs and/or histological and immunohistological investigations were utilized in several studies [12,31,32,33,35]. In the research conducted by Miyazaki and coworkers, [34] his team used microcomputed tomography in addition to histological and histochemical analysis.

### 3.3. Risk of Bias

The findings of the evaluation of the risk of bias are reported in Table 4. Five studies were judged to have a high risk of bias [12,32,34,35,36], and two were judged to have an unclear risk of bias [31,33]. Concerning the order of allocation, five of them were found to have a high risk of bias, while the rest had an unclear risk of bias. Considering allocation concealment and assessors’ blinding, the majority of the trials had an unclear risk of bias. Information on the randomization of animal housing was also unclear. The risk of bias connected to the animal random selection for the outcome evaluation was assessed to be unclear for all of them as well. Regarding the processing of incomplete data and the reporting of selective outcomes, the risk of bias was graded as unclear for all of them.

## 4. Discussion

### 4.1. Summary of Evidence

Mandibular asymmetry can be caused by developmental alterations such as condylar agenesis, hypoplasia, or hyperplasia; acquired abnormalities such as trauma, tumors, infections, or functional mandibular displacement; and other local factors that can all cause functional mandibular displacement [2]. The objective of this study was to gather and evaluate all the published animal studies that examined how masseter muscle resection impacted mandibular growth. Seven articles were short-listed, as the focus was studies with radiographic macroscopic outcomes. In two papers [12,34], the sole technique of intervention was the unilateral complete removal of the masseter muscle, whereas the bilateral complete removal of the masseter muscle was only carried out in four investigations [31,32,35,36]. In the study conducted by Horowitz et al. [33], there were two groups with the masseter completely removed: one unilaterally and the other bilaterally. When the masseter muscle is impaired unilaterally, the skull deviates to one side, as shown by the research of Horowitz et al. [33]. This abnormality develops when the masseter muscle is affected, either by direct surgical removal or by loss of the zygomatic arch. All the studies that met the inclusion criteria included both control and experimental groups, which were compared to one another to determine statistically significant changes. Insufficient detail was provided in the description of the statistical approaches. As a result, the quality of the present systematic review’s findings may be compromised by inadequate or erroneous data that resulted in systemic errors.

Asymmetries in the maxilla were also discovered by the cephalometric evaluations. When comparing the two sides of the masseter muscle separation, the length of the maxilla and mandible was found to be asymmetrical; however, this asymmetry was not observed between the groups. In addition, the results of the cephalometric assessments demonstrated that in the groups that underwent removal and dissection, there was a statistically significant difference between the two sides for each measurement. These measurements comprised the distance between the tympanic bulla and the mesial root of the first molar (TB-MR), the distance between the tympanic bulla and the infraorbital foramen (TB-IF), and the distance between the infraorbital foramen and the incisal point (IF-IP) [12] These investigations showed that the unilateral chewing patterns brought on by idiopathic disorders or asymmetric functioning of the masseter muscles might result in mandibular asymmetries brought on by a shorter mandible and an atrophic angular process. Several of them focused mostly on histology and biochemical findings, even though radiography is the diagnostic tool of choice in daily dental practice.

Microcomputed tomography performed by Miyazaki et al. [34] revealed the asymmetric development of the mandible in the experimental group and degenerative alterations in the resected side condyles. In the same study, histological analysis revealed that the central part of the resected side had considerably less total cartilage than the unresected side. These findings align with those published by Sakurai et al. [36] who examined disc thickness in the experimental group and found that it was much lower than in the control group.

Our examination focused on past studies utilizing rats as the main experimental model. Higher financial expenses limit testing with non-human primates, and ethics forbid enlisting humans as experimental subjects in procedures that may have irreversible or unwanted repercussions. Since mandibular growth is correlated with overall growth and changes with chronological age, the age of the animals is crucial. Studies involving animals going through a growth spurt were the focus of this review. The animals’ ages, which ranged from 21 days [35], 30 days [33], 3 weeks [32,36], and 4 weeks in the study by Rodrigues et al. [12] to 5 weeks by Miyazaki et al. [34] were explicitly indicated. Monje et al. [31] conducted the longest range of experiments, from 30 to 130 days. Bone turnover is regulated by sexual hormones, and both genetic and environmental factors control the pace and pattern of mandibular growth. The study conducted by Yonemitsu et al. [32] demonstrated that bilateral masseter excision in prepubertal rats impaired mandibular growth in adulthood. After 9 weeks, there was a considerable reduction in the disc thickness across all regions but mainly in the intermediate zone, where the experimental group participated. In addition, Monje et al. [31] could not find any statistically significant changes in the macroscopic, morphometric, or histological characteristics between the control and sham-operated groups. Ιn the study conducted on both growing and adult rats, they exhibited a verticalization of the face and a posterior–inferior rotation of the jaw. The results from the immunohistochemical study by Yonemitsu et al. [32] demonstrated that the type I collagen staining in the discs of the experimental group was much less intense than that of the control group. The inferior rotational pattern was found to be characterized by a downward and forward inclination of the occlusal plane, as well as an increase in the mandibular plane angle, according to the findings of Navarro et al. [35]. According to their findings, the condyle’s development is adaptive at least in terms of direction, since it tends to stay in the same relative location to the fossa. The rotational pattern of rat face growth, especially mandibular growth, is influenced by changes in the masticatory muscle environment, according to the findings of this study [35].

Lastly, our research revealed that the removal of the masseter muscle in developing rats caused a considerable reduction in the length of the jaw that extended to the maxilla [12]. The observed alterations of the various mandibular regions may be related to the remodeling produced by a weak masticatory force system, resulting in altered mandibular growth. The development of the condyles, maxillary sutures, and dentoalveolar processes contributes to the growth-related displacement of the jaw. The eventual result was a shortened mandible with an atrophying angular process.

### 4.2. Strengths and Limitations

Our systematic review was conducted in accordance with well-established guidelines, which are described in the Materials section. There is a relatively limited amount of data due to the nature of study included in this review and the characteristics of the data. Few studies employed X-rays or CBCT to evaluate mandibular growth in rats, while some studies used histological markers. Therefore, more research is needed to identify radiographic approaches for assessing mandibular growth in rats. Considering the preceding data and their characteristics, we are willing to assume that the methods explored in experimental animal studies appear to have a substantial impact on the vertical rotation of the mandible caused by alterations in the angular process that are atrophic, as well as asymmetry in the maxilla and mandibular shortening.

## 5. Conclusions

The masseter muscle is one of the main muscles responsible for mandibular function and plays a crucial role in mandibular development. The muscle is also involved in maintaining the shape and position of the mandible. The removal of the masseter muscle can potentially affect the mandibular morphology through several mechanisms. Firstly, it can lead to changes in muscle forces, which may alter the position and orientation of the mandible. Secondly, it can affect bone remodeling, leading to alterations in the shape and structure of the mandible. Lastly, the removal of the masseter muscle can affect the growth and development of the mandible, leading to changes in its size and shape. Despite the importance of the masseter muscle in mandibular development and function, there are still gaps in our understanding of the underlying mechanisms. For example, it is not clear how changes in muscle forces translate into alterations in mandibular morphology. Additionally, the role of other muscles in mandibular development and function needs to be further investigated. 

This paper aimed to contribute to our understanding of the relationship between masseter muscles and craniofacial morphology by providing a comprehensive review of the existing literature in this field. By synthesizing and analyzing previous research findings, this paper highlighted gaps in our knowledge and identified areas for further investigation.

The results of this study showed that the masseter muscle significantly affects the craniofacial growth and development. There is a substantial decrease in the sagittal and vertical growth of the mandible in rats with masseter muscle excision. In addition, the excision of the masseter muscle affects the mandibular morphology, including the condylar region and the angle and the direction of growth of the mandible. Future research could focus on developing more sophisticated models to study the effects of the masseter muscle on mandibular morphology. This could involve the use of imaging techniques to measure the changes in bone structure and the development of computational models to simulate the effects of muscle forces. Additionally, more studies are needed to investigate the long-term effects of masseter muscle removal on the mandibular development and function. Understanding the relationship between the masseter muscles and craniofacial morphology has important clinical applications. For example, orthodontic and surgical interventions that aim to correct malocclusions and other craniofacial abnormalities can benefit from a deeper understanding of this relationship. Additionally, research in this area may contribute to the development of therapies for conditions that affect the function of the masticatory system, such as temporomandibular joint disorders (TMDs). Ultimately, a better understanding of this relationship could lead to improved patient outcomes and quality of life.

## Figures and Tables

**Table 1:** The eligibility criteria for this systematic review.

Domain	Inclusion Criteria	Exclusion Criteria
Participants	Rats of any age, sex, and strain that had masseter muscle resection.	Other species than rats.
Intervention	Rats that underwent unilateral or bilateral masseter muscle resection.	Rats undergoing a variety of interventions, such as denervation or resection of the masseter muscles, the removal or grinding of teeth, insertion of devices to alter mastication, and modification of food consistency.
Outcomes	Quantitative macroscopic data regarding masseter muscle resection measured mainly by radiography [lateral cephalometric radiographs, Cone Beam CT, micro-CT, etc.].	Histological or histochemical assessments [i.e., histologic immunohistochemistry] regarding treatment immunohistochemistry.
Study design	Experimental prospective controlled studies (according to the Scottish Intercollegiate Guidelines Network algorithm for classifying study design)	Non-comparative studies. Reviews, systematic reviews, and meta-analyses.

**Table 2:** Strategy for database search.

Database	Search Strategy	Hits
**PubMed** http://www.ncbi.nlm.nih.gov/pubmed (accessed on 31 October 2022)	(monkey* OR rat OR rats OR mouse OR mice OR murine OR rabbit*) AND (((resection OR detachment OR “surgical detachment” OR dissection OR removal OR manipulation OR “muscle manipulation” OR atrophy) AND (masseter OR temporalis OR pterygoid)) OR masseterectomy)	483
**Scopus** https://www.scopus.com/search/form.url?zone=TopNavBar&origin=searchbasic (accessed on 31 October 2022)	TITLE-ABS (monkey* OR rat OR rats OR mouse OR mice OR murine OR rabbit*) AND (((resection OR detachment OR “surgical detachment” OR dissection OR removal OR manipulation OR “muscle manipulation” OR atrophy) AND (masseter OR temporalis OR pterygoid)) OR masseterectomy)	212
**Web of Science™** https://www.webofscience.com (accessed on 31 October 2022)	TITLE: (monkey* OR rat OR rats OR mouse OR mice OR murine OR rabbit*) AND (((resection OR detachment OR “surgical detachment” OR dissection OR removal OR manipulation OR “muscle manipulation” OR atrophy) AND (masseter OR temporalis OR pterygoid)) OR masseterectomy) Timespan: all years; search language = English	151

**Table 3:** Features of the included reports. CG: control group, EG: experimental group, F: female, M: male, h: hour, d: day, m: month, w: week, masseter muscle resection (MR), temporal muscle resection (TR), suprahyoid muscle resection (SR).

Article	Sample	Intervention	Comparison	Method of Assessment	Results
Rodrigues et al., 2009 [12]	30 F, 4 w old, Wistar rats	Unilateral complete removal of the masseter muscle was performed in the EG1.	Ten rats in each group. EG1: Unilateral complete removal of the masseter muscle. EG2: Masseter muscle detachment and repositioning were carried out in the dissection group. EG3: The sham-operated group underwent surgical access.	Cephalometric radiograph.	In the removal group, the jaw was deviated to the right, whereas the dissection group exhibited distinct facial asymmetry. The removal and dissection groups had substantial differences between sides for all measurements: tympanic bulla to mesial root of the first molar (TB-MR), tympanic bulla to infraorbital foramen (TB-IF), and infraorbital foramen to incisal point (IF-IP). In the sham-operated group, however, there were no substantial differences between sides for all measurements.
Monje et al., 1994 [31]	140 M, age range from 30 d to 130 d, Wistar rats	Bilateral complete removal of the masseter muscle was performed in the EG1.	EG1: Bilateral complete removal of the masseter muscle. EG2: Mock operation. CG: Control group.	Lateral cephalometric X-ray. Histological analysis.	Muscular resection rotated the mandible posteriorly, altering the TMJ articulation.
Yonemitsu et al., 2007 [32]	36 M, 3 w old, Wistar rats	Bilateral complete removal of the masseter muscle was performed in the EG.	EG (n = 20): Bilateral complete removal of the masseter muscle. CG (n = 16): Control group.	Lateral cephalometric X-ray. Immunohistology.	EG: The lateral X-rays tended to have large mandibular plane angles. EG: Rat condyles had thinner chondroblastic layers than CG. EG: IGF-1r immunopositive cells expressed substantially less than CG cells. EG condylar bone had more TRAP-positive cells and chondrocytes.
Horowitz et al., 1955 [33]	24, 30 d old, Sherman strain rats (They do not mention the sex of the rats)	Unilateral complete removal of the masseter muscle was performed in the EG1. Bilateral complete removal of the masseter muscle was performed in the EG2.	EG1 (n = 9): Unilateral complete removal of the masseter muscle. EG2 (n = 3): Bilateral complete removal of the masseter muscle. CG (n = 12): Control group.	Lateral cephalometric X-ray.	Unilateral removal of the masseter caused significant skeletal changes to the skull, jaws, and dental arches. The rostrum shifted to the unoperated side, and the mandible warped inferiorly and laterally. Bilateral masseter muscle removal did not result in any gross dental abnormalities. Following unilateral masseter muscle removal in rats, functional imbalance in the remaining masticatory muscles may cause architectural changes in the craniofacial skeleton.
Miyazaki et al., 2016 [34]	40, 5 w old, Wistar rats (They do not mention the sex of the rats)	The masseter muscle was unilaterally completely removed in the EG.	Twenty rats in each group. EG: Unilateral complete removal of the masseter muscle. CG: Control group.	Microcomputed tomography. Histological and histochemical examination of the condyle.	Microcomputed CT indicated asymmetric development in the EG, and the resected side condyles showed degenerative alterations. Histological investigation revealed that the total cartilage in the central area of the resected side in both the EG and CG was substantially thinner than in the non-resected side. Compared to the CG, asporin expression was considerably greater on the resected side and much lower on the unresected side. TGF-b1-immunopositive cells were substantially more abundant in the non-resected side than in the resected side or the CG.
Navarro et al., 1995 [35]	35 F, 21 d old, Wistar rats	Bilateral complete removal of the masseter muscle was performed in the EG1.	EG1 (n = 8): Bilateral complete removal of the masseter muscle. EG2 (n = 8): Bilateral complete removal of the temporal muscle. EG3 (n = 8): Bilateral complete removal of suprahyoid muscles. CG (n = 11): Control group. The results were evaluated at two time periods: 42 (prepubertal) and 60 days after birth (pubertal rats).	Lateral cephalometric X-ray. Histological examination of the condyle. Dentoalveolar processes height measurement.	Two patterns of mandibular rotation were discovered: superior after TR and inferior after MR. A less pronounced superior rotational pattern was detected following SR. Morphologic alterations were greater in older rats. Younger rats had more condylar histologic alterations. Articular growth mostly occurred at the maxillary and mandibular dentoalveolar processes. The condylar growth rate was modifiable to a limited degree. Condylar direction: The inferior mandibular rotation pattern created an upward rotational pattern of the upper viscerocranium.
Sakurai et al., 2007 [36]	30 M, 3 w old, Wistar rats	Bilateral complete removal of the masseter muscle was performed in the EG.	Fifteen rats in each group. EG: Bilateral complete removal of the masseter muscle. CG: Control group.	Measurement of the TMJ disc thickness. Immunohistology.	Compared to the CG, the EG had thinner discs in each region. The experimental group found type I collagen and decorin at the front and posterior bands of the disc, while the CG found it throughout the disc. EG had considerably fewer IGF-1r immunopositive cells than CG.

**Table 4:** Risk of bias for the following studies The following signaling questions were assessed for each study: 1: Was the allocation sequence adequately generated and applied?; 2: Were the groups similar at baseline or were they adjusted for confounders in the analysis?; 3: Was the allocation adequately concealed?; 4: Were the animals randomly housed during the experiment?; 5: Were the caregivers and investigators blinded to the intervention that each animal received?; 6: Were the animals selected at random for outcome assessment?; 7: Was the outcome assessor blinded?; 8: Were incomplete outcome data adequately addressed?; 9: Are the reports of the study free of selective outcome reporting?; 10: Was the study apparently free of other problems that could result in a high risk of bias?

Studies	1	2	3	4	5	6	7	8	9	10	Summary
Rodrigues et al., 2009 [12]	High	Low	Unclear	Unclear	Unclear	Unclear	Unclear	Unclear	Low	Unclear	High
Monje et al., 1994 [31]	Unclear	Unclear	Unclear	Unclear	Unclear	Unclear	Unclear	Unclear	Low	Unclear	Unclear
Yonemitsu et al., 2007 [32]	High	Unclear	Unclear	Unclear	Unclear	Unclear	Unclear	Unclear	Low	Unclear	High
Horowitz et al., 1955 [33]	Unclear	Unclear	Low	Unclear	Unclear	Unclear	Unclear	Unclear	Low	Unclear	Unclear
Miyazaki et al., 2016 [34]	High	Unclear	Unclear	Unclear	Unclear	Unclear	Unclear	Unclear	Low	Unclear	High
Navarro et al., 1995 [35]	High	Unclear	Unclear	Unclear	Unclear	Unclear	Unclear	Unclear	Low	Unclear	High
Sakurai et al., 2007 [36]	High	Unclear	Unclear	Unclear	Unclear	Unclear	Unclear	Unclear	Low	Unclear	High

## Data Availability

The data presented in this study are available in the included studies of this systematic review.
